# Integrating and optimizing genomic, weather, and secondary trait data for multiclass classification

**DOI:** 10.3389/fgene.2022.1032691

**Published:** 2023-03-29

**Authors:** Vamsi Manthena, Diego Jarquín, Reka Howard

**Affiliations:** ^1^ Department of Statistics, University of Nebraska-Lincoln, Lincoln, NE, United States; ^2^ Agronomy Department, University of Florida, Gainesville, FL, United States

**Keywords:** genomic selection, classification, multi-omics, sparsity, data integration

## Abstract

Modern plant breeding programs collect several data types such as weather, images, and secondary or associated traits besides the main trait (e.g., grain yield). Genomic data is high-dimensional and often over-crowds smaller data types when naively combined to explain the response variable. There is a need to develop methods able to effectively combine different data types of differing sizes to improve predictions. Additionally, in the face of changing climate conditions, there is a need to develop methods able to effectively combine weather information with genotype data to predict the performance of lines better. In this work, we develop a novel three-stage classifier to predict multi-class traits by combining three data types—genomic, weather, and secondary trait. The method addressed various challenges in this problem, such as confounding, differing sizes of data types, and threshold optimization. The method was examined in different settings, including binary and multi-class responses, various penalization schemes, and class balances. Then, our method was compared to standard machine learning methods such as random forests and support vector machines using various classification accuracy metrics and using model size to evaluate the sparsity of the model. The results showed that our method performed similarly to or better than machine learning methods across various settings. More importantly, the classifiers obtained were highly sparse, allowing for a straightforward interpretation of relationships between the response and the selected predictors.

## 1 Introduction

Modern plant breeding programs are collecting an increasing amount of data of various types from several sources such as multiple secondary phenotypic traits (other than the main trait of interest), high-throughput phenotyping data, weather data, hyper-spectral images, and different types of -omics data such as genomics, transcriptomics, proteomics, metabolomics, *etc.* It is believed that many secondary phenotypic traits are often positively associated with the main trait, a fact that most prediction models for genomic selection (GS) do not take advantage of. Given the availability of different data mentioned above, an important question is how these various data types could be integrated to improve prediction. Integrating different data types becomes a complex challenge when the data types have very different dimensions. In the case of GS, the high-dimensional nature of the genomic data is well known. Genomic data are often found to be in the form of Single Nucleotide Polymorphisms (SNPs), which can range from the thousands to millions. On the other hand, data types such as secondary traits can be fewer than 20. Naive concatenation of various data types into existing GS models could lead to poor results because of the differing sizes. Genomic variables would out-compete all other variable types in terms of explaining the variation in the response due to their sheer numbers. A key challenge in such scenarios is to build models that are able to access the unique information presented in each data type to improve the prediction capabilities.

Early attempts at integration of data types for GS show promising results (Schrag et al., 2018). Lopez-Cruz et al. (2020) proposed the concept of a penalized selection index, where the selection index (SI) was linear combinations of high-dimensional secondary traits that maximized the correlation between the primary trait and the secondary traits. The SI was used in a G-BLUP model as a covariate or using bivariate methods with SI and main trait as the two responses. Arouisse et al. (2021) examined the possibility of other dimensionality reduction methods such as penalized regression and random forests to reduce the dimension of the secondary trait set and used them in bivariate or multivariate settings. Sandhu et al. (2021) explored the possibility of including all secondary traits along with the main trait in multivariate GS methods and hence their approach was optimal in the presence of a small number of secondary traits.

Another underdeveloped area of research involves developing models for non-Gaussian phenotypic traits. Crop yield, a continuous trait (can be modeled with a Gaussian distribution), is often the most important trait that plant breeding programs want to improve. Some other continuous traits that breeders focus on include quality (shape, size, and other aesthetic qualities), time to maturity, plant height, and seed weight. Sometimes, breeders are also interested in improving categorical phenotypic traits such as resistance to drought or salinity, susceptibility to disease, and days to maturity or flowering. While extensive literature covers the prediction of continuous traits, there is limited literature developing GS models for classification.

Since the seminal work of (Meuwissen et al., 2001), genomic selection (GS) models harnessed the genomic marker information combined with observed phenotypic data to improve the prediction of unobserved phenotypic values. Most of the GS methods proposed over the last 2 decades were developed for continuous phenotypic traits, which were assumed to be normally distributed (Kizilkaya et al., 2014; Montesinos-López et al., 2015a; Montesinos-López et al., 2017; Silveira et al., 2019). However, several crops have categorical traits that have agronomic importance (Iwata et al., 2013; Martínez-García et al., 2017; Sousa et al., 2019). These categorical traits could be binary or multicategory in nature. Some examples of such traits include resistance to disease, resistance to salinity, degree of infection, fruit quality, fruit external appearance, fruit size, and the number of reproductive nodes.

When the number of categories in the response is large, and the data follows an approximately normal distribution, treating the response as normal may be reasonable. However, if the number of categories is small and has a well-defined ordering among them, the normality approximation leads to biased estimates of the mean and the variance components (Stroup, 2012; Stroup et al., 2018). Generalized linear mixed models (GLMMs) were proposed as the most suitable alternative to model the response variable according to the appropriate distribution it arose from.

Unfortunately, GLMMs are not directly implementable in GS due to the high dimensionality of the genomic data, where the number of predictors is far greater than the number of observations. Bayesian GLMM approaches were proposed to address the high-dimensionality problem and the multicollinearity issue prevalent in genomic data. Some popular methods include BayesA (Meuwissen et al., 2001), BayesB (Meuwissen et al., 2001), Bayes C*π* (Habier et al., 2011), Bayesian ridge regression and Bayesian LASSO (Park and Casella, 2008). Wang et al. (2013) extended the Bayes A, Bayes B, and Bayes C*π* using threshold models (Gianola, 1982) to estimate categorical traits in animal breeding. Following this idea, Montesinos-López et al. (2015a) extended the genomic best linear unbiased predictor (GBLUP) model (Burgueño et al., 2012; Jarquín et al., 2014) for ordered categorical data using a probit link function. They also introduced a logit link based model for categorical traits that included interaction effects (Montesinos-López et al., 2015b; Montesinos-López et al., 2017). While some of the models above were developed to predict ordinal categorical traits based on genomic information, none of the models had provisions to integrate multi-type data to improve prediction.

High-dimensional prediction is the most prevalent challenge in the GS problem and has been an active area of research. Feature selection is a common strategy to reduce the dimensionality to perform such predictions. Feature selection also helps remove irrelevant features and improve the interpretability of the final model. Variable selection methods such as forward selection, backward selection, and best-subset selection were popular but performed poorly in high-dimensional data (James et al., 2013). Penalization methods such as ridge regression (Hoerl and Kennard, 1970), LASSO (Tibshirani, 1996), elastic net (Zou and Hastie, 2005), and their variants were proposed for feature selection and work in high-dimensional data as well. We describes some of the relevant advancements of these penalization methods next.

Ghosal et al. (2009) proposed a novel method called forward iterative regression and shrinkage technique (FIRST) that combined forward selection with penalization methods such as LASSO to predict a continuous response. FIRST effectively combined two categories of feature selection methods. Turnbull et al. (2013) proposed a new method called selection technique in orthogonalized regression models (STORM), that acted as an extension to FIRST. Their method was developed especially for the case of highly correlated predictors. Both FIRST and STORM had lower errors than the traditional LASSO, especially when the predictors were correlated with each other. More importantly, their methods also led to a smaller final model than LASSO, allowing for greater interpretability of the relationships between the predictors and the response. FIRST and STORM methods could be very useful methods for GS because they work well in the presence of correlated predictors, which is a common issue with genomic data.

FIRST and STORM methods were developed for a continuous response and were regression methods. Ghosal et al. (2016) proposed a penalized forward selection for the support vector classification method (CLASSIC) which was a forward selection based SVM for high-dimensional classification. Their models led to lower error rates than traditional SVM along with providing significantly leaner models. The method also has low memory requirements from a computational perspective. While FIRST, STORM and CLASSIC dealt with high-dimensional predictions, they did not provide solutions for combining data types.

Jarquin et al. (2022) (in review) combined the idea of sparse prediction and classification from these papers and applied them to the context of combining two data types for GS. They incorporated secondary traits, which represented a low-dimensional data set, and genomic information, which was a high-dimensional component. Their method used a penalized forward selection based logistic regression inspired by high-dimensional prediction models such as the FIRST, STORM, and CLASSIC. They showed that their method provided sparse models with favorable classification accuracy compared to standard ML methods such as RF, SVM, boosting, and linear discriminant analysis (LDA). Model sparsity refers to the number of variables used for the classification task and hence the smaller the model size, the greater the sparsity. Greater sparsity in the final models allows for easier interpretation of relationships between predictors and response, as well as determining important variables.

Additionally, global climate change and extreme environmental changes are an inescapable reality of the day, presenting an escalating challenge to food production worldwide. Resilience and adaptability among crops are essential to ensure food security. Resilience refers to the stability of the yield in the face of extreme weather conditions, while adaptability refers to how crops react to changes in environments (Macholdt et al., 2020). Understanding the impact of environmental covariates on the phenotypic outcome will help select crops that are better suited across environments and select the best-performing varieties for specific environments. In this work, a Finlay-Wilkinson (FW) regression (Finlay and Wilkinson, 1963) based approach was used to find the optimal window where the weather had the greatest impact on the phenotypic traits of interest. Similar approaches to finding optimal windows of time for weather information have been the subject of recent research (Li et al., 2018; Millet et al., 2019; Guo et al., 2020; Costa-Neto et al., 2021; Li et al., 2021). However, these studies did not focus on integrating data types for GS.

To summarize, this paper focused on three challenges to improve GS: integrating multiple data types, optimizing weather data to improve forecasting, and developing methods for high-dimensional forecasting for categorical phenotypic traits. We developed a three-stage method to integrate three data types (secondary traits, weather data, and genomic data) of differing dimensionality to predict a binary categorical phenotypic trait. The main goal of our method is to integrate genomic, weather, and secondary trait information to classify a trait of interest which can be modeled as a binary variable. Binary variables have two classes, such as diseased or not, fraud or not, resistant or not, *etc.* The practical implication of our method is that plant breeders can collect different data types with significantly different dimensions and have a way to combine them for the purpose of predicting traits that have two possible outcomes. We also employed techniques to extend this method for multi-class categorical traits (traits that express as more than two classes). Furthermore, FW regression was implemented as a pre-processing step to the three-stage method, to identify optimal time windows to optimize the weather data and improve the interpretability of the effect of weather on the main trait. This enables plant breeders to include weather information that influences the trait of interest instead of all available weather information. It has a practical implication by learning which growing stages impact the prediction. Finally, the performance of the proposed methods was compared to two standard machine learning (ML) methods - random forests (RF) and support vector machines (SVM).

The rest of the paper is organized as follows. First, we present a short overview of relevant literature that motivated our proposed three-stage method. Our proposed method for binary traits was presented in the Materials and Methods section. Next, we describe details about the strategy that allows our method to handle multi-class traits as well as a description of how FW regression was implemented. This section ends with a discussion of the metrics used to evaluate the classification ability of the methods for binary as well as multi-class traits. The details of the real data set used to demonstrate our methods were described next. Then, we present the results of the method and compare them to other standard methods in terms of their performance and finally conclude with a discussion and future directions.

## 2 Materials and methods

Penalized approaches (Hoerl and Kennard, 1970; Tibshirani, 1996; Zou and Hastie, 2005; James et al., 2013) for regression and classification are common in the presence of high-dimensional data. However, a classical penalized logistic regression approach does not work in our context because it does not allow for variables of certain data types to be considered for the model building before others. Combining data types of disparate sizes invites the risk of the “crowding-out” issue discussed earlier. Outnumbering often leads to out-competing, resulting in the lower-dimensional data being disregarded in the final model. In a naive concatenation approach, it is plausible that none of the secondary traits or weather variables are retained in the final model. In order to avoid this, a forward selection approach was considered, whereby we included variables one at a time. Forward selection also allowed control of the order in which data types are considered. By first considering low-dimensional data types, this method ensured that the classifier used all information available to explain the variability in the primary trait before allowing higher-dimensional data types to explain the variation in the response.

Another issue was that the genomic and weather variables also impacted the secondary traits. Before considering all the variables in the model building, the effect of genomic and weather variables had to be removed from the secondary traits and their true intrinsic effects had to be obtained. Isolating the intrinsic effect of the secondary traits also ensured that the potential effect of genomic or weather variables was not mistakenly ascribed to the secondary traits. Separating the effects also allowed for a simpler and cleaner interpretation of variable importance and relationships between the response and the explanatory variables.

In the first stage of the modeling, we computed the intrinsic effect of the secondary traits devoid of the weather and genomic effects. In order to remove the genomic and weather effects, the weather variables were first regressed on each of the secondary traits to obtain the residuals. Then, the genomic variables were regressed on the secondary traits to obtain another set of residuals. In the second stage of the modeling, we used a training data set to build a logistic regression classifier combined with a penalized forward-selection scheme to include phenotypic residuals into the model before allowing weather variables and finally allowing genomic variables. Through the iterative process of the forward selection scheme, only the most influential predictor was selected to enter the model at each step. The third and final stage of the proposed method concentrated on improving the classification through a threshold search process. Traditionally, a threshold of *p* = .5 is used to categorize the predicted probabilities obtained from a logistic classifier, where an observation with a probability greater than .5 is classified as a 1 and below .5 as a 0. However, when the number of 1s and 0s in the binary response are significantly different, often referred to as class imbalance, the threshold of .5 may lead to poor classification accuracy. Hence, we used an optimization data set to determine the optimal threshold to improve classification accuracy in this third stage of modeling. Finally, the coefficients obtained from the second stage of modeling and the optimal threshold obtained from the third modeling stage were used to predict the class assignment in the test data set and the corresponding results were used to evaluate the model’s performance.

### 2.1 Three-stage method for binary classification

In this section, we build on the overview presented above by describing all the pertinent details to implement the proposed method for GS. Let the binary main trait of interest be represented by *y*_*i*_, the two sets of residual for the secondary traits be denoted by ${\hat{u}}_{i}=\left({\hat{u}}_{iW_{1}},{\hat{u}}_{iW_{2}},\ldots ,{\hat{u}}_{iW_{P}},{\hat{u}}_{iV_{1}},{\hat{u}}_{iV_{2}},\ldots ,{\hat{u}}_{iV_{P}}\right)$ , the weather covariates be denoted by *w*_*i*_ = (*w*_*i*1_, *w*_*i*2_, *…* , *w*_*iQ*_), and the genomic variables be denoted by *v*_*i*_ = (*v*_*i*1_, *v*_*i*2_, *…* , *v*_*iR*_), where *i* = 1, 2, *…* , *n*. Without loss of generality, let us assume that *E*(*U*) = *E*(*W*) = *E*(*V*) = 0 and Var(*U*) = *I*_*P*_, Var(*W*) = *I*_*Q*_, and Var(*v*) = *I*_*R*_, where *U* = (*u*_1_, *u*_2_, *…* , *u*_*n*_), *V* = (*v*_1_, *v*_2_, *…* , *v*_*n*_), and *W* = (*w*_1_, *w*_2_, *…* , *w*_*n*_). Essentially, we replaced the variables with their standardized versions.

The first stage of the method involved evaluating the two sets of residuals of the secondary traits by removing the effects of weather and genomic covariates. A penalized regression model was used to compute the residuals of each *u*_*ip*_, where *p* = 1, 2, *…* , *P* and *i* = 1, 2, *…* , *n*. First, we regressed the weather variables on each of the secondary traits *u*_*ip*_ and obtained the residuals ${\hat{u}}_{iW_{p}}=u_{ip}-w_{i}^{T}{\hat{b}}_{p}$ , where ${\hat{b}}_{p}=\left({\hat{b}}_{p1},{\hat{b}}_{p2},\ldots ,{\hat{b}}_{pQ}\right)$ were the corresponding regression coefficients and $w_{i}^{T}$ was the vector of weather covariates associated *i*th observation. The regression coefficients were estimated by minimizing the penalized sum of squares:

$$\overset{n}{\underset{i=1}{\sum }}{\left(u_{ip}-w_{i}^{T}b_{p}\right)}^{2}+\lambda \overset{Q}{\underset{q=1}{\sum }}\text{pen}\left(\left|b_{pq}\right|\right),$$ (1)

where the penalty function can be any of the standard penalty functions such as the LASSO, adaptive LASSO, or ridge regression penalties. Ridge regression uses a *L*_2_-penalty with the residual sum of squares (RSS) loss function to shrink the coefficients associated with predictors towards zero. However, due to the nature of the *L*_2_-penalty, regularization is performed, but none of the coefficients are set exactly to zero. LASSO model, on the other hand, uses a *L*_1_-penalty with the RSS to shrink the coefficients and sets some coefficients to exactly zero, effectively performing feature selection. Elastic net is a compromise between ridge regression and LASSO and uses a linear combination of both *L*_1_ and *L*_2_ penalties. Thus, an elastic net has the advantages of regularization and feature selection. Adaptive LASSO (Zou, 2006) was proposed as an alternative to LASSO in the presence of high multicollinearity among explanatory variables, which is seen commonly in genomic data sets. We compared the various penalty functions, including the raw residuals with zero penalty, to determine which yielded the best results. The entire process was repeated by regressing the set of genomic variables on each of the secondary traits to obtain the residuals ${\hat{u}}_{\mathrm{iV p}}=u_{ip}-v_{i}^{T}{\hat{d}}_{p}$ , where ${\hat{d}}_{p}=\left({\hat{d}}_{p1},{\hat{d}}_{p2},\ldots ,{\hat{d}}_{pR}\right)$ were the corresponding regression coefficients and $v_{i}^{T}$ was the vector of genomic variables associated *i*th observation.

After obtaining the intrinsic effect of the secondary traits, represented by the two sets of residuals obtained in step one, we moved on to the second stage of the method. Here, the penalized forward selection was implemented to ensure that the secondary trait residuals were entered first into the model, followed by the weather covariates, and finally followed by the genomic variables. This approach ensured that higher-dimensional data types did not crowd out the smaller-dimensional data types and improved how the variables included in the model explained the variability in the response. We used a logistic classifier as the model structure for its simplicity and ease of interpretability. The probability mass function (PMF) of the *C*-class multinomial logistic classifier for class *c* is given by:

$$P\left(Y_{i}=c|\Theta \right)=\frac{e^{\sum_{t=1}^{T}\theta_{ct}z_{it}}}{1+\sum_{c^{\prime }=1}^{C-1}e^{\sum_{t=1}^{T}\theta_{c^{\prime }t}z_{it}}},$$ (2)

where Θ = (*α*_*cW1*_, *…* , *α*_*cWP*_, *α*_*cV1*_, *…* , *α*_*cVP*_, *β*_*c*1_, *…* , *β*_*cQ*_, *γ*_*c*1_, *…* , *γ*_*cR*_) is a *C* × *T* matrix of coefficients associated with the predictors and $z_{i}=\left(z_{i1},z_{i2},\ldots ,z_{iT}\right)=\left({\hat{u}}_{\mathrm{iW1}},\ldots ,{\hat{u}}_{\mathrm{iWP}},{\hat{u}}_{\mathrm{iV 1}},\ldots ,{\hat{u}}_{\mathrm{iV P}},w_{1},\ldots ,w_{Q},v_{1},\ldots ,v_{R}\right)$ represents the vector of all predictor values for observation *i*. Here, *T* = 2*P* + *Q* + *R*. The classification for a new test observation is given by:

$$\hat{c}=\arg\underset{c}{\max}P\left(Y_{\left(n+1\right)}=c|\hat{\Theta }\right),$$ (3)

where $\hat{\Theta }$ is the set of coefficient estimates obtained from the Newton-Raphson estimation method with a LASSO penalty.

#### 2.1.1 Newton-Raphson (NR) method

Using the PMF function defined in Eq. 2, the log-likelihood function required for the NR method is given by:

$$\begin{array}{c} f\left(\theta_{ct}|S\right)=\underset{i:y_{i}=c}{\sum }\log\left(\frac{e^{\sum_{t=1}^{T}\theta_{ct}z_{it}}}{1+\sum_{c^{\prime }=1}^{C-1}e^{\sum_{t=1}^{T}\theta_{c^{\prime }t}z_{it}}}\right)+\underset{i:y_{i}\neq c}{\sum }\log\left(\frac{e^{\sum_{t=1}^{T}\theta_{ct}z_{it}}}{1+\sum_{c^{\prime }=1}^{C-1}e^{\sum_{t=1}^{T}\theta_{c^{\prime }t}z_{it}}}\right), \end{array}$$ (4)

where *S* = {(*y*_1_, *z*_1_), *…*, (*y*_*n*_, *z*_*n*_)} denoted the set of observations with *y*_*i*_ representing the response and *z*_*i*_ representing the vector of predictors associated with the *i*th observation. In the presence of penalty terms for each of the data types, a modified version of Eq. 4 can be maximized as:

$$f\left(\theta_{ct}|S\right)-\lambda_{1}|\alpha_{cp}|-\lambda_{2}|\beta_{cq}|-\lambda_{3}|\gamma_{cr}|,$$ (5)

where the *λ*’s are the penalty values. Here, we used a LASSO-based penalization which ensured that several coefficients were set to zero, making the model more sparse. Sparsity allowed for greater interpretability of the final model because there were only a few predictors with non-zero coefficients. Using the second-order Taylor series approximation, the coefficients were updated in the (*k*+1)^th^ NR iteration as follows:

$$\theta_{ct}^{k+1}\left(L\right)=\theta_{ct}^{k}-s\frac{f^{\prime }\left(\theta_{ct}^{k}\right)+\max\left(\lambda_{1},\lambda_{2},\lambda_{3},0\right)}{f^{\prime \prime }\left(\theta_{ct}^{k}\right)}$$ (6)

$$\theta_{ct}^{k+1}\left(R\right)=\theta_{ct}^{k}-s\frac{f^{\prime }\left(\theta_{ct}^{k}\right)-\max\left(\lambda_{1},\lambda_{2},\lambda_{3},0\right)}{f^{\prime \prime }\left(\theta_{ct}^{k}\right)},$$ (7)

where *s* is the step size, also known as the learning rate. More specifically, the NR iteration updates for each of the different data types were given by the following equations:

$$\begin{array}{llll} \alpha_{cp}^{k+1}\left(L\right) & =\alpha_{cp}^{k}-s\frac{f^{\prime }\left(\theta_{ct}^{k}\right)+\lambda_{1}}{f^{\prime \prime }\left(\theta_{ct}^{k}\right)} & \alpha_{cp}^{k+1}\left(R\right) & =\alpha_{cp}^{k}-s\frac{f^{\prime }\left(\theta_{ct}^{k}\right)-\lambda_{1}}{f^{\prime \prime }\left(\theta_{ct}^{k}\right)}\\ \beta_{cq}^{k+1}\left(L\right) & =\beta_{cq}^{k}-s\frac{f^{\prime }\left(\theta_{ct}^{k}\right)+\lambda_{2}}{f^{\prime \prime }\left(\theta_{ct}^{k}\right)} & \beta_{cq}^{k+1}\left(R\right) & =\beta_{cq}^{k}-s\frac{f^{\prime }\left(\theta_{ct}^{k}\right)-\lambda_{2}}{f^{\prime \prime }\left(\theta_{ct}^{k}\right)}\\ \gamma_{cr}^{k+1}\left(L\right) & =\gamma_{cr}^{k}-s\frac{f^{\prime }\left(\theta_{ct}^{k}\right)+\lambda_{3}}{f^{\prime \prime }\left(\theta_{ct}^{k}\right)} & \beta_{cq}^{k+1}\left(R\right) & =\beta_{cq}^{k}-s\frac{f^{\prime }\left(\theta_{ct}^{k}\right)-\lambda_{3}}{f^{\prime \prime }\left(\theta_{ct}^{k}\right)}. \end{array}$$

Here, *L* and *R* represented the left- and right-derivatives of Eq. 5 with respect to *θ*_*ct*_. Following the optimization solution provided in Wu et al. (2009), if $\theta_{ct}^{k+1}\left(L\right)<0$ , then we set $\theta_{ct}^{k+1}=\theta_{ct}^{k+1}\left(L\right)$ and if $\theta_{ct}^{k+1}\left(L\right)>0$ , we set $\theta_{ct}^{k+1}=\theta_{ct}^{k+1}\left(R\right)$ . If either $\theta_{ct}^{k+1}\left(L\right)=0$ or $\theta_{ct}^{k+1}\left(R\right)=0$ , then we set $\theta_{ct}^{k+1}=0$ . The iteration process continued until the convergence criteria were met.

#### 2.1.2 Algorithm

The likelihood associated with the logistic regression was intractable, and hence NR iterative methods were used to obtain the coefficients associated with the predictors. In this section, we presented the algorithm used to initialize and update the coefficients in each iteration of the NR method as well as the stopping criterion. The algorithm was by setting *θ*_*ct*_ = 0 for all *c* and *t*. Suppose we denote the *k*th penalized log-likelihood from Eq. 5 as PLL_*m*_(*c*, *t*).

1. Update each *θ*_*ct*_ using the NR update rules from Eqs 6, 7. Continue iterations until:

$$|PLL_{m}\left(c,t\right)-PLL_{m+1}\left(c,t\right)|\leq \epsilon |PLL_{m+1}\left(c,t\right)+1|,\text{and}$$ (8)

$$PLL_{m}\left(c,t\right)\leq PLL_{m+1}\left(c,t\right).$$ (9)

2. The NR updates start with a step size *s* = 1. If the $\theta_{ct}^{m+1}$ does not satisfy Eq. 8 or Eq. 9, we repeat the procedure using *s* = 1/2, 1/2^2^, 1/2^3^, *…* , 1/2^10^. If the PLL does not improve with changing *s*, we set $\theta_{ct}^{m+1}=\theta_{ct}^{m}$ .
3. Stop the iteration process when no variable is selected.

In the proposed algorithm, there were five hyperparameters that need to be tuned, {*s*, *ϵ*, *λ*_1_, *λ*_2_, *λ*_3_}. We started with step size *s* = 1 as a reasonable value for the parameter and varied it by halving it successively until the convergence criteria were met. The *ϵ* value was set to be 10^–3^ for the secondary traits, 10^–5^ for the weather traits, and 10^–8^ for the genomic variables traits. The choice of these *ϵ*′s was another way to give more importance to the data types with smaller dimensions as well as ensure greater sparsity of the final model and hence, can be varied to suit the objectives of the problem. We did not observe any changes in predictive power by increasing the *ϵ*′s to 10^–8^ for all the data types. A cross-validation grid-search was used to find the optimal values of *λ*′s by testing various combinations of the *λ*′s ranging from 1 to 10. For each combination of *λ*′s, we estimated the predictor coefficients and then evaluated the models using various classification metrics. The optimal combination corresponded to the one with the best classification metrics.

### 2.2 FW regression to optimize weather information

One of the primary objectives of this work was to find the most sparse models that had the best classification performance. While the relationships between the selected secondary trait variables and the response or the genomic variables and the response were easy to interpret, the relationship between the weather covariates and the response was more challenging to understand. Data on four weather variables were collected daily over the entire growing season of 100 days, yielding 400 weather covariates. The variables were maximum temperature (Tmax), minimum temperature (Tmin), wind speed (WS), and rainfall (Precip). Weather covariates that led to the best classifier were selected without regard for the interpretation of the individual covariates chosen. For instance, suppose the three weather covariates chosen were WS at day 45, Tmax at day 18, and Tmin at day 72. There is no insight into what these individual days mean for a breeder or a farmer and how to determine the practical significance of these covariates.

An alternate approach could be to select windows of time when the selected set of weather covariates have the most impact on the main trait. For instance, suppose we select day 18 to day 25 as the window of time in the season. Then, we could include all daily weather covariates for those days in our three-stage model to understand the impact of the window on the response. Such windows of time allow for greater interpretability of the impact of weather conditions during the growing season on the final plant production. Further, extreme weather conditions in the identified windows of time could help forecast potential losses, and farmers could take actions to mitigate losses, if possible.

Plant breeding programs often collect data on several weather variables such as wind speed, humidity, daylight hours, temperature, *etc.* When several weather variables are present, a different optimal time window can be obtained for each one of them. However, including a separate set of covariates for each optimal time window in our model increases the model size. Weather variables can be combined to form a single environmental index as an alternative. In this work, we proposed using principal component analysis (PCA) to combine the weather variables and extracted the first principal component as a singular environmental index. PCA method ensured that information across the different weather variables was combined. The first PC corresponds to the linear combination of variables that explains the maximum variability present in the weather data. When daily weather data is available, PCA can be performed daily. All the weather variables for a day were combined to form the first PC for that day. This process was repeated to obtain the first PCs every day in the growing season. Following this, the average of PCs was computed within each time window to represent the environmental effect of the time window. Normalization of the weather variables is necessary before PCA is applied to ensure that all variables are on a similar scale and did not disproportionately skew the PC calculations.

Suppose the main trait can be expressed as follows:

$$y_{ij}=\mu +G_{i}+E_{j}+e_{ij},$$ (10)

where *y*_*ij*_ represented the value of the main trait for genotype *i* in environment *j* and *e*_*ij*_ represented the random error, *i* = 1, 2, *…* , *t* and *j* = 1, 2, *…* , *k*, where *t* was the number of genotypes and *k* was the number of environments. Then, the environmental means for the *j*th environment is represented by:

$${\bar{y}}_{.j}=\mu +\frac{1}{t}\overset{t}{\underset{i=1}{\sum }}G_{i}.$$ (11)

The time window mean of weather variables is represented as follows:

$${\bar{w}}_{pj}^{\left(b,e\right)}=\frac{1}{\left(e-b\right)}\overset{e}{\underset{d=b}{\sum }}w_{\mathrm{pjd}},$$ (12)

where, *w*_*pjd*_ denoted the value of the *p*th weather variable in *j*th environment on day *d* and ${\bar{w}}_{pj}^{\left(b,e\right)}$ was the mean of the weather variables in the time window. Then, *b* represented the beginning day of the time window, and *e* represented the ending day. Here, *w*_*pjd*_ could also be the first PC of the linear combination of all the weather variables available for the day *d*. Finally, the linear association between the environmental means and the mean of the time window was defined as the R-squared value when simple linear regression is performed between ${\bar{y}}_{.j}$ and ${\bar{w}}_{pj}^{\left(b,e\right)}$ . For simple linear regression, the R-squared value is simply the square of the correlation between the variables. Hence, the R-squared was computed as:

$${\left(\rho_{pj}^{\left(b,e\right)}\right)}^{2}={\left(\text{cor}\left({\bar{y}}_{.j},{\bar{w}}_{pj}^{\left(b,e\right)}\right)\right)}^{2},$$ (13)

where, $\rho_{pj}^{\left(b,e\right)}$ represented the correlation between the environmental mean of the *j*th environment and the mean of the (*b*, *e*)-th time window for the *p*th weather variable. R-squared values range between 0 and 1, with 1 indicating perfect linear association and 0 indicating no linear association. Hence, the optimal window corresponds to the window with the highest R-squared found using the method described above.

We found the optimal time window for the weather variables as a pre-processing step. Hence, we included all the weather variables within the optimal time window and did not perform any feature selection for this data type in the modeling stage. We set *λ*_2_ = 0 in the penalized likelihood function Eq. 5 and obtained the following reduced penalized likelihood function:

$$f\left(\theta_{ct}|S\right)-\lambda_{1}|\alpha_{cp}|-\lambda_{3}|\gamma_{cr}|.$$ (14)

The rest of the three-stage method remained the same as in section 2.1. The NR updating algorithm also remained identical. Here too, we used a cross-validation grid search to find the optimal values of *λ*_1_ and *λ*_3_.

### 2.3 Extending our method for multi-class classification

Predicting a response with two classes is known as a binary classification problem. Some popular supervised learning methods for binary classification include logistic regression, naive Bayes, decision trees, random forests, support vector machines, and neural networks (Kotsiantis et al., 2006; James et al., 2013). These methods also handle multi-class problems in one of two ways. The first approach involves modifying the relevant algorithm to extend to multi-class settings, and the second involves deconstructing the multi-class problem into a set of binary classification problems, known as binarization strategies (Galar et al., 2011).

Binarization strategies are prevalent because they allow simpler ways to form decision boundaries separating the two classes. Binarization allows for a greater choice of classification algorithms because almost every classification algorithm such as logistic regression, SVM, neural networks, *etc.* was introduced initially for binary classification. It has also been established that the performance of a single multi-class classifier is no better than an aggregation of a set of binary classifiers (Sánchez-Marono et al., 2010; Galar et al., 2011). There are two major ways to perform binarization: one-vs-one (OVO) and one-vs-all (OVA) (Lorena et al., 2008; Sánchez-Marono et al., 2010; Galar et al., 2011; Abramovich et al., 2021).

The OVO method has better predictive ability than the OVA in general (Rifkin and Klautau, 2004; Pawara et al., 2020). The performance of the OVA approach is similar to OVO when the base classifier is well-tuned (Rifkin and Klautau, 2004). However, the performance of OVA suffers in the presence of class imbalance (Galar et al., 2011). On the other hand, the main advantage of OVA is that the number of binary classifiers required is in the order of *K* while the number of binary classifiers for OVO is in the order of *K* ^2^. Thus, the number of classifiers required for OVO increases exponentially as a function of the number of classes in the response. For example, for a response with ten classes, OVO requires 45 classifiers, whereas OVA requires only ten classifiers.

In this study, we extended our methods to deal with a categorical response with three classes by opting for the OVA binarization. Since the number of classes was three, both OVO and OVA required the same number of binary classifiers - three. The objective of our proposed method was a multi-class classification for an ordinal categorical response with any number of classes. Hence, we chose the OVA approach because of computational frugality as well as generalizability. Further details about OVO and OVA can be found in section 2 of the Supplementary Material.

For a *K* class response variable, the OVA approach creates *K* binary classification problems. Suppose we have a response with three classes. Then, for the first binary classifier, OVA sets all the response values of class 1 as 1 and the other two classes as 0. In the second binary classifier, class 2 is set as 1 and rest as 0, and the third classifier has class 3 set as 1 and rest as 0. For each binary sub-problem, any classifier can be used for the classification task. In this study, we used a modification of the three-stage method developed earlier as the classifier of choice. The proposed method was a penalized logistic regression with forward selection. The output from this classifier was a vector of probabilities that an observation belonged to class 1. While many standard classifiers output probabilities like logistic regression, some classifiers such as neural networks output a non-probability based score. A softmax activation function can be used to convert the scores to a probability when using other classifiers (Pawara et al., 2020).

Using the OVA approach for a three-class problem, we obtained a set of three probabilities for each observation corresponding to the probability that the observation belonged to each of the three classes. The predicted class was simply the class with the highest probability for each observation, referred to as the maximum probability approach.

#### 2.3.1 Reason for not using optimal threshold step

For three-class classification, we obtained three probabilities for each observation coming from the three binary classifiers. Class assignments depended only on comparing these probabilities and picking the class with the maximum probability. Thus, unlike traditional binary classification, the class assignment was independent of any threshold. Hence, we dropped the threshold search step from the method proposed in the binary classification algorithm and used the rest of the method as the classifier for multi-class classification.

We tried to improve the classification by searching for optimal thresholds between class 1 vs rest and class 3 vs rest. The idea was to partition the probability space into three regions corresponding to the three classes, based on these two thresholds. However, the approach had poor forecasting performance, especially for class 2. A possible reason was that three completely separate classifiers were used, each with its own set of coefficients associated with the predictors leading to three sets of linear predictors that had significant overlaps in the predictor space. Thus, this approach was not feasible, and the best performance was observed when using the maximum probability approach.

### 2.4 Classification metrics

**FIGURE 1:**
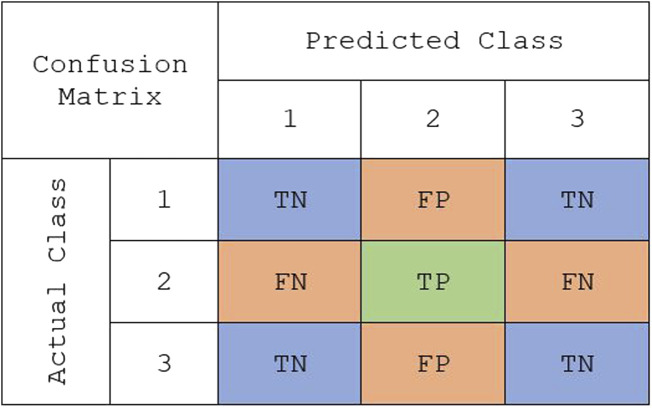
A representative confusion matrix representing the true class vs. predicted class for “class 2” in a multiclass classification: true positive (TP), false positive (FP), true negative (TN), and false negative (FN).

Binary classification metrics can be modified to make them suitable for multi-class problems. For multi-class classification, the performance was assessed using the following metrics: overall accuracy, macro true positivity rate (TPR), and macro true negativity rate (TNR). Overall accuracy is a metric that remains the same between binary and multi-class problems. It is defined as the total number of correctly classified observations out of the total number of observations in the data set. In binary classification, TPR and TNR are easily defined because there is one positive and one negative class. However, these metrics need to be modified when there are multiple classes. Instead, TPR and TNR can be computed for each class, and then the weighted mean could be found to compute the weighted macro TPR and weighted macro TNR. The weights are determined by the proportions of each class in the training data set. Using the sample multi-class confusion matrix in Figure 1, TPR and TNR for class *k* can be defined as:

$${\text{TPR}}_{k}=\frac{TP}{TP+FN}$$ (15)

$${\text{TNR}}_{k}=\frac{TN}{TN+FP},$$ (16)

where TP was the number of true positives, TN was the number of true negatives, FP was the number of false positives, and FN was the number of false negatives. Then, the weighted macro TPR (mTPR) and TNR (mTNR) for *K* classes are given by:

$$\text{mTPR}=\frac{w_{1}\times TPR_{1}+w_{2}\times TPR_{2}+\cdots +w_{K}\times TPR_{K}}{K}$$ (17)

$$\text{mTNR}=\frac{w_{1}\times TNR_{1}+w_{2}\times TNR_{2}+\cdots +w_{K}\times TNR_{K}}{K},$$ (18)

where, *TPR*_*k*_ refers to the TPR for the *k* ^th^ class, *w*_*k*_ denotes the proportion of observations in class *k* in the training data, and *w*_1_+⋯ + *w*_*K*_ = 1.

### 2.5 Data

We used a chickpea data set to evaluate the performance of the proposed model and contrast it with standard machine learning methods such as random forest (RF) and support vector machines (SVM). The data set contained phenotypic, weather, and genomic SNP data. The phenotypic and weather data were collected at four locations, namely International Center for Agricultural Research in the Dry Areas (ICARDA) - Amlaha, International Crops Research Institute for the Semi-Arid Tropics (ICRISAT) - Patancheru, Junagadh Agricultural University (JAU) - Junagadh, and Rajasthan Agricultural Research Institute (RARI)—Durgapura, over two seasons, 2014–15 and 2015–16. Genotypic information was available for *n* = 749 lines and the corresponding phenotypic information was available for all the lines across the eight environments (location by year combinations).

Over 15 phenotypic variables were collected across the eight environments. However, only seven of those were available in all eight environments. We selected only these as the primary and secondary phenotypic traits for the analysis. The seven phenotypic variables were days to flowering (DF), plant height (PLHT), basal primary branch (BPB), basal secondary branch (BPB), days to maturity (DM), pods per plant (PPP), and 100 seed weight (100-SDW). This work focused on days to maturity (DM) as the primary trait of interest. The rest of the six traits form the secondary trait data type.

The second data type in this study was the weather data collected daily over the entire growing season of 100 days. Depending on the location, the growing season was between October and April. Several weather variables were collected at each location; however, only four variables were commonly present across the four locations for each day. The variables were maximum temperature, minimum temperature, wind speed, and rainfall. Since the four weather traits were collected daily over 100 days, there were a total of 400 weather variables that form the weather data type.

Genomic Single nucleotide polymorphisms (SNP) data was available for the 749 lines. We randomly selected 10,000 markers for each line to form the genomic data type. To summarize, there were six secondary traits, 400 weather traits, and 10,000 SNPs as the final set of predictor variables. We considered a three-class DM variable as the response for the multi-class classification demonstration and also implemented the proposed three-stage method for a binary trait, whose implementation and results can be found in section 3 of the Supplementary section.

### 2.6 Multi-class trait implementation

**FIGURE 2:**
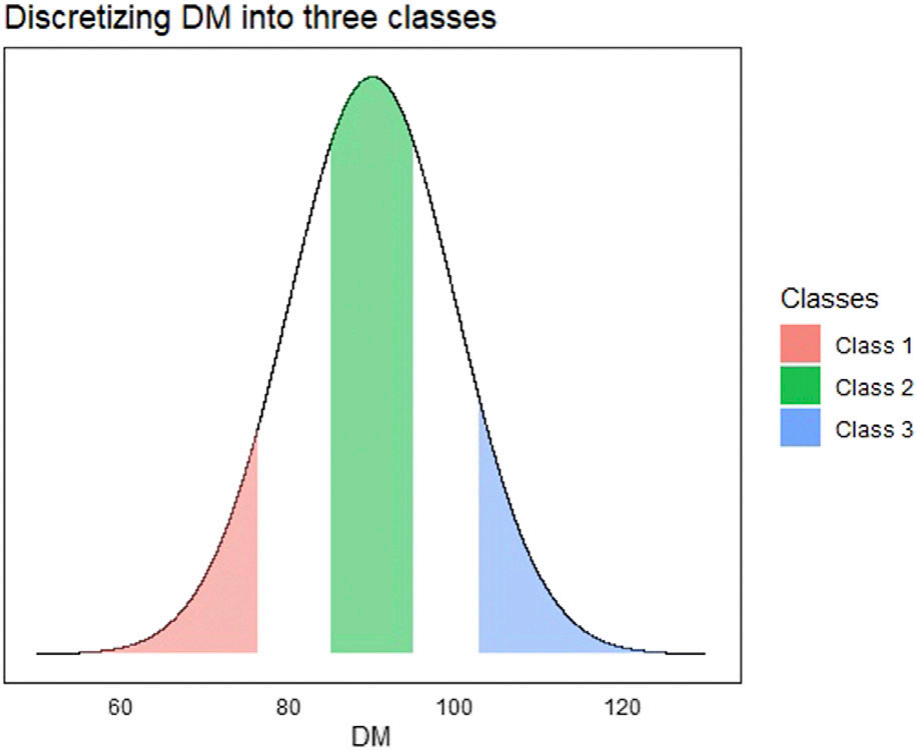
Visualization depicting the process of discretizing a continuous trait (Days to Maturity—DM) into a multi-class trait. All observations in red are denoted as class 1, green as class 2, and blue as class 3.

For the multi-class implementation, we created a three-class categorical response using the DM trait that was continuous. The DM trait was discretized into three levels corresponding to low, average, and high days to maturity. For the continuous trait DM, all observations in the bottom 25th percentile were denoted as class 1 and top 25th percentile as class 3. We also found a 25th percentile centered around the mean of the continuous trait and denoted that as class 2. The discretization of the continuous response is depicted in Figure 2.

The performance of the multi-class methods was evaluated using three different class-balance settings: 33-34-33, 40-40-20, and 10-80-10. 33-33-33 refers to the balanced class setting, while 10-80-10 refers to the extreme imbalance setting where 80% of the observations are in class 2% and 10% in each of the other two classes. We randomly sampled 280 observations to create the datasets with different class ratios. For each class ratio, 20 replications were created and averaged the performance across the replications to avoid sampling bias.

We decomposed the multi-class problem into a set of binary classification problems using the OVA approach. As discussed earlier, threshold searching methods were not employed due to this OVA approach. Thus, the 280 observations were split into a train and test set in the ratio of 200/80. The training data set was used for the penalized logistic regression modeling step, and the test data set was used to evaluate the performance of the models.

The penalization effect was evaluated with the help of a baseline model *G* + *E* + *P* that did not have a penalty applied to any of the data types denoted by MC0. Here, *G* refers to the genomic data, *E* refers to the weather data, and *P* refers to the secondary trait data. We evaluated the cases where all the secondary traits *PenG* + *PenE* + *P* and penalized the secondary traits *PenG* + *PenE* + *PenP* were included, where *Pen* refers to penalized versions of the data types. These models are denoted as MC1 and MC2, respectively. The optimal time window was applied to optimize the weather data and evaluated the following models *PenG* + *FWE* + *P* (MT1) and *PenG* + *FWE* + *PenP* (MT2), where *FWE* refers to the optimized weather data. Finally, these models were compared to the RF and SVM machine learning models. Table 1 summarizes all the model settings that were evaluated in this paper, along with the model notations.

**TABLE 1:** Summary of models assessed in this paper for a multi-class categorical trait.

Model	Notation	*λ*_1_	*λ*_2_	*λ*_3_
G + E + P	MC0	0	0	0
PenG + PenE + P	MC1	0	varying	varying
PenG + PenE + PenP	MC2	varying	varying	varying
PenG + FWE + P	MT1	0	0	varying
PenG + FWE + PenP	MT2	varying	0	varying
Support Vector Machines	SVM	–	–	–
Random Forest	RF	–	–	–

## 3 Results

In our research, we focused on several aspects of developing models to improve breeding and aid the selection process. The focus was to integrate three different data types with differing dimensions for the purpose of predicting phenotypes that can be categorized. In addition, we wanted to see whether a subset of the weather information is sufficient to achieve the same prediction accuracy as we can reach with the full set of weather information.

**FIGURE 3:**
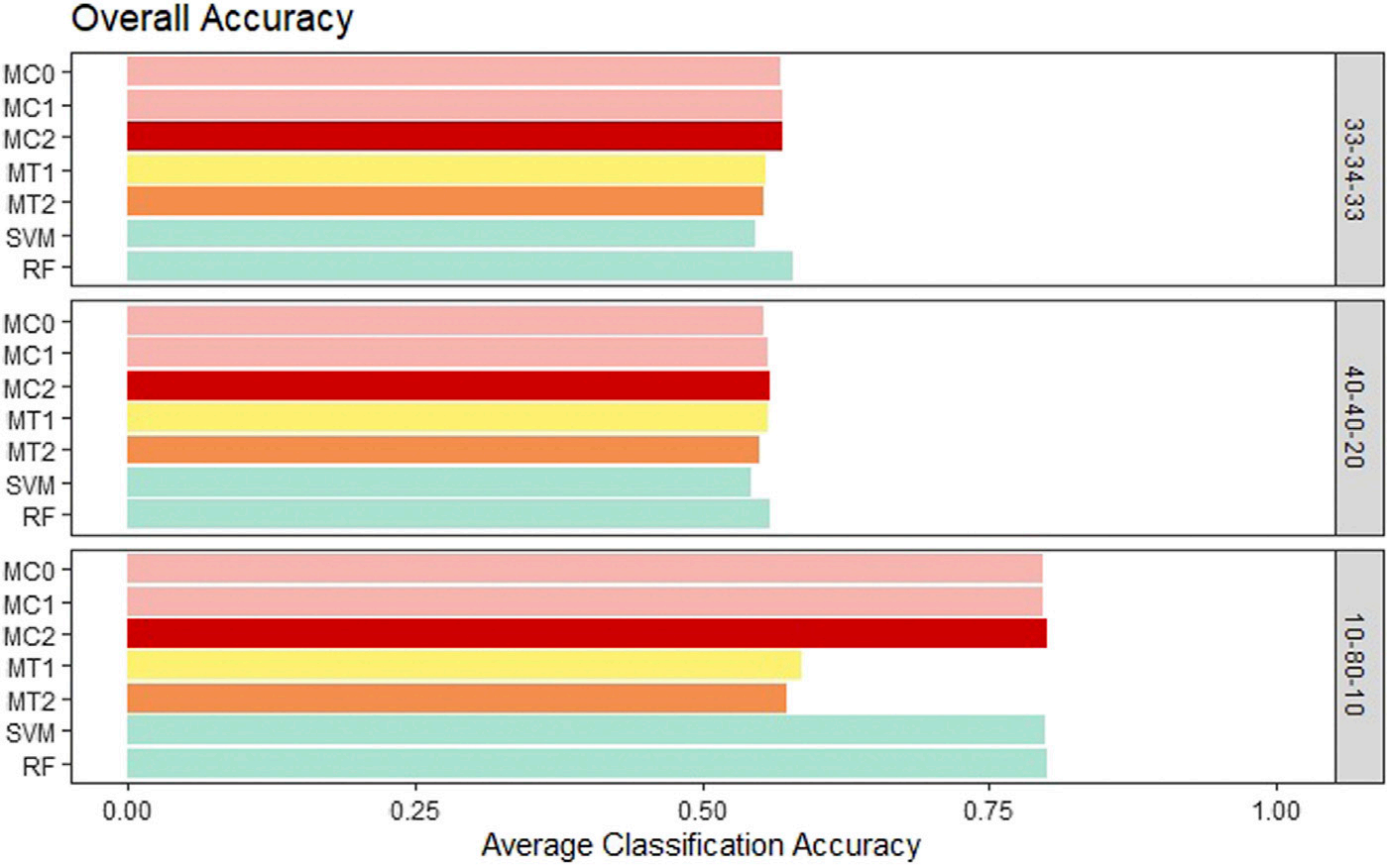
Bar plot comparing the seven different classification models based on overall classification accuracy averaged over the 20 replications within each class balance setting for the Days to Maturity (DM) trait with three classes. The seven models and their notations are as follows: G + E + P (MC0), PenG + PenE + P (MC1), PenG + PenE + PenP (MC2), PenG + FWE + P (MT1), PenG + FWE + PenP (MT2), support vector machine (SVM), and random forest (RF).

First, the proposed models were evaluated in terms of overall accuracy, and we also included TPR and TNR. Seven classification models (MC0, MC1, MC2, MT1, MT2, SVM, RF) were evaluated and compared. We wanted to see whether the penalization-based methods and methods that incorporate the reduced amount of weather information can outperform machine learning techniques. Also, we wanted to determine whether the model performance is influenced by the rate of the imbalance in the data. All the proposed models showed similar performance to ML models for the balanced class and the medium imbalance settings (40–40–20). All the models had an overall accuracy of ∼.55 in both these settings. The penalization-based models (MC0, MC1, and MC2) had similar overall accuracy to the optimal time window based models (MT1 and MT2) for these two settings. However, in the extreme imbalance class of 10-80-10, MT1 and MT2 had significantly worse accuracy of .57 and .59 instead of the .80 accuracies for the penalization-based and ML models. It is important to note that both the ML models and the penalization models ended up predicting all observations as class 2 for the 10-80-10 case and hence resulted in an accuracy of 80%, matching the proportion of class 2 in that setting. Weighted macro TPR and TNR metrics provide better insight into a model’s predictive ability in the presence of imbalance, and hence we looked at these metrics next. Across the board, the standard error associated with the average of the overall accuracy, mTPR, and mTNR were in the order of 10^–3^ to 10^–4^ and hence are not presented here. Overall accuracy results for all the models can be seen in Figure 3.

**FIGURE 4:**
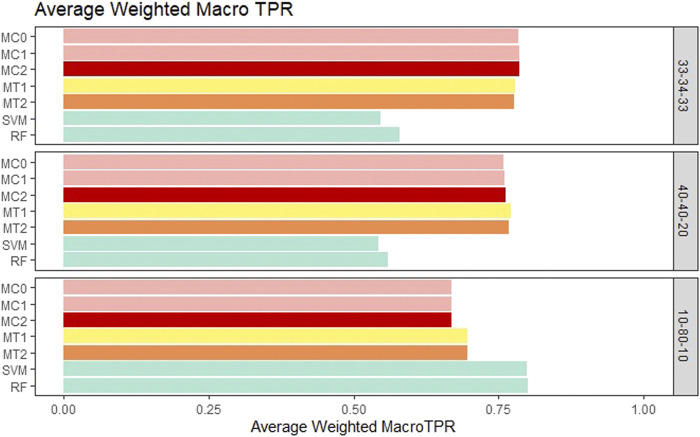
Bar plot comparing the seven different classification models based on the weighted macro true positivity rate (TPR) averaged over the 20 replications within each class balance setting for the Days to Maturity (DM) trait with three classes. The seven models and their notations are as follows: G + E + P (MC0), PenG + PenE + P (MC1), PenG + PenE + PenP (MC2), PenG + FWE + P (MT1), PenG + FWE + PenP (MT2), support vector machine (SVM), and random forest (RF).

**FIGURE 5:**
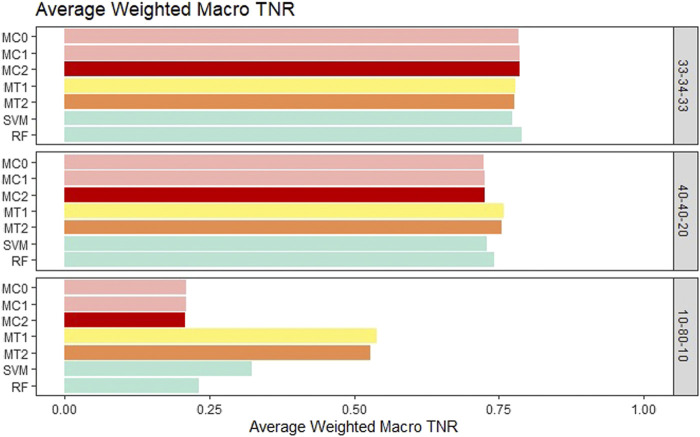
Bar plot comparing the seven different classification models based on the weighted macro true negativity rate (TNR) averaged over the 20 replications within each class balance setting for the Days to Maturity (DM) trait with three classes. The seven models and their notations are as follows: G + E + P (MC0), PenG + PenE + P (MC1), PenG + PenE + PenP (MC2), PenG + FWE + P (MT1), PenG + FWE + PenP (MT2), support vector machine (SVM), and random forest (RF).

For the balanced class setting, the weighted macro TPR values for the proposed models were around .78, while the ML models had values around .58. This represents a 20% higher TPR value for our proposed models. On the other hand, macro TNR values for the proposed models were similar to the ML methods in the balanced case. We saw similar trends for the medium imbalance case as well. However, in the extreme imbalance case, the ML methods outperformed the proposed methods in terms of the weighted macro TPR metric. In contrast, MT1 and MT2 had ∼25% higher macro TNR than the penalization based and ML models. These results can be viewed in Figure 4 and Figure 5. The proposed models had similar or better performance in the balanced and medium imbalanced class settings for both the mTPR and mTNR metrics. In the extremely imbalanced class, there was a tie between the proposed methods being better in terms of mTNR and ML being better in terms of mTPR.

**FIGURE 6:**
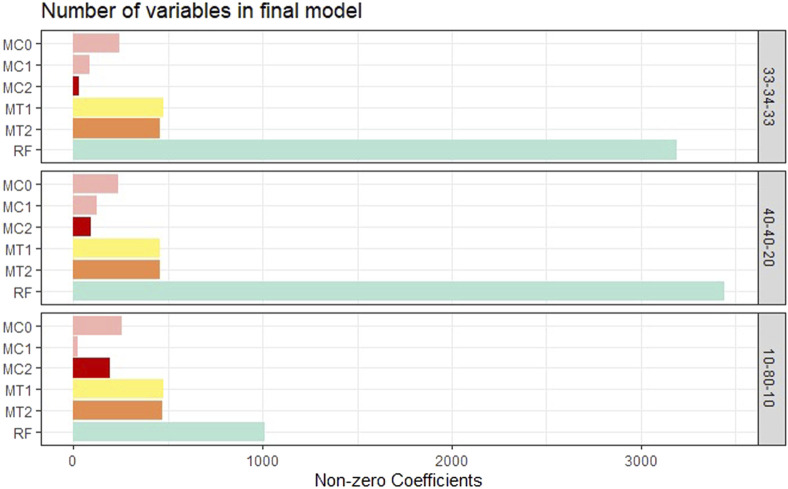
Bar plot comparing the proposed models to random forest based on model size averaged over the 20 replications within each class balance setting for the Days to Maturity (DM) trait with three classes. The six models and their notations are as follows: G + E + P (MC0), PenG + PenE + P (MC1), PenG + PenE + PenP (MC2), PenG + FWE + P (MT1), PenG + FWE + PenP (MT2), and random forest (RF).

Model interpretability was one of the primary objectives of this study along with the evaluation of the model’s predictive power. Model interpretability can be assessed by evaluating the complexity of the model used. Generally, including more predictors into the model results in a more complex model that can lead to difficulty in interpretability. Our methods showed a tremendous improvement over the ML methods in model sizes. Across all class balance settings, our method had close to 90% fewer predictors in the final models compared to the ML models. The penalized methods had smaller model sizes between the penalization-based and the optimal window-based approaches. This complements what we observed with the binary trait results (presented in the supplementary materials). MC1 and MC2 had the smallest models consistently across the class settings. Just as with the binary trait, the penalization-based methods MC0, MC1, and MC2 did not include any genomic variables in the final model, while MT1 and MT2 did. Refer to Figure 6 as well as Supplementary Figures S9, S10 (in supplementary materials) for visualizations corresponding to the model size comparisons. All the metric comparisons between the seven models are presented in Table 2.

**TABLE 2:** Summary of results for the seven different models across the three different class-balance settings. The performance was measured using overall accuracy (Acc), weighted macro true positivity rate (mTPR), weighted macro true negativity rate (mTNR), and model size (MDS). The seven models and their notations are as follows: G + E + P (MC0), PenG + PenE + P (MC1), PenG + PenE + PenP (MC2), PenG + FWE + P (MT1), PenG + FWE + PenP (MT2), support vector machine (SVM), and random forest (RF).

Balance	Model	Acc	mTPR	mTNR	MDS
33—34—33	MC0	.57	.78	.78	240.5
	MC1	.57	.78	.79	83.8
	MC2	.57	.79	.79	28.5
	MT1	.56	.78	.78	476.9
	MT2	.55	.78	.78	459.9
	SVM	.55	.55	.77	–
	RF	.58	.58	.79	3191.6
40—40—20	MC0	.55	.76	.72	238.8
	MC1	.56	.76	.73	60.3
	MC2	.56	.76	.73	45.2
	MT1	.56	.77	.76	460.3
	MT2	.55	.77	.74	458.6
	SVM	.54	.54	.73	–
	RF	.56	.56	.74	3443.8
10—80—10	MC0	.80	.67	.21	253.2
	MC1	.80	.67	.21	24.3
	MC2	.80	.67	.21	48.7
	MT1	.59	.70	.54	475.9
	MT2	.57	.70	.53	468.4
	SVM	.80	.80	.23	–
	RF	.80	.80	.32	1013.4

## 4 Discussion

One of the recent challenges in plant breeding and especially in terms of accelerating genetic gain is how to utilize all the data that are collected. With high-throughput technologies, information is collected on the molecular level, on the different environments including weather information, and factors that describe the different management practices. This system of diverse information has the potential to connect the different genotypes in different environments and explain how they might be related and ultimately benefit the prediction of traits of interest.

This paper proposed a novel three-stage classification method to improve multi-class classification when combining multi-type data. More specifically, we developed a classification method for binary and multi-class responses using secondary trait data, weather covariates, and marker information. We used the one-versus-all (OVA) binarization approach (Galar et al., 2011; Abramovich et al., 2021) to decompose the multi-class problem into a set of binary classification problems and aggregated the results using a maximum probability approach (each observation). The method was evaluated in different settings, including various penalization schemes and class balances, and compared with standard machine learning methods. Various metrics (Acc, mTPR, mTNR) were used to evaluate classification accuracy and model size to evaluate the sparsity of the model. Overall, our model showed excellent promise in predictive ability. Our proposed models matched or outperformed ML methods across almost all settings and metrics. Most importantly, the classifiers obtained through our models were highly sparse. Specifically, MC1 and MC2 models used fewer than 80 predictors to obtain similar performance to ML methods. This greatly increases the ability for manual dissection of the relationships between individual predictors and the multi-class trait.

The improved performance of the proposed model, as compared to the ML methods, can be attributed to the manner in which the stages of the proposed method were constructed. First, by isolating the intrinsic effect of the secondary traits, we reduced the confounding effects. Reducing confounding helped separate the effect of each data type on the response as well as helped improve the independence between data types. It is well known that collinearity and high-correlated predictors significantly harm prediction and classification efforts. Secondly, by controlling the order in which data types entered the model, we gave the secondary traits the best chance of being selected in the final model. This process ensured that weather and genomic variables were not selected unless they significantly enhanced the model and ensured that the secondary traits were not ignored just because of their low dimensionality. We further exacerbated this effect by the choice of *ϵ*′s for each data type. Secondary traits are sometimes collected during the early- or mid-season, and hence our model allows breeders to estimate the end-season main trait better based on this information. Finally, penalization in conjunction with forward selection played a fundamental role in reducing model sizes, thereby allowing breeders to make data-driven decisions based on the relationships at play. The main advantage of our method for plant breeders is that they can leverage genomic, weather, and secondary trait data to classify the trait of interest. We showed a method for incorporating different data types that they can potentially collect in a way that enables them to select lines for advancements more efficiently.

One of the key strengths of the proposed method is its modular nature. In the first stage, we used ridge-based penalized regression to extract the intrinsic effect of the secondary traits devoid of weather and genomic effects. The ridge penalty can be substituted for any other penalties such as LASSO, adaptive LASSO, elastic net, adaptive elastic net, *etc.* The choice of penalty depends on the data set at hand, its unique characteristics, and the study’s objectives. In the second stage of the method, we used a penalized forward-selection based logistic regression for the model building. We used a LASSO-based penalty in this stage for the feature selection advantages offered by LASSO. LASSO can again be substituted for one of the other penalty functions based on the application. Logistic regression was selected due to its simplicity and interpretability. If interpretability is not one of the concerns, this could be switched out for one of the other, more complex classification methods available, including the machine learning algorithms. The high degree of modularity lends flexibility to the model. We believe that it will allow the method to have a wide range of applicability to any problem that involves combining data types of different sizes.

There are several immediate extensions that we can foresee. In this work, we considered 10,000 randomly selected SNPs as the genomic data type. Surprisingly none of these SNPs were selected in the final models based on the proposed method, which could be a result of the random selection process of these SNPs from a much larger pool of available SNPs. Alternatively, it could also be attributed to measures employed to keep model sizes as small as possible such as the choice of penalties and *ϵ* values in the stopping criteria of the NR method. Given that secondary traits and weather covariates explained a large proportion of the response, computational resources are wasted to evaluate the genomic data type’s impact. Variable screening methods (Fan and Fan, 2008; Fan and Lv, 2008; Wang, 2009; Hao and Zhang, 2014; Liu et al., 2015) are fast and crude methods to reduce the dimensionality of ultra-high dimensional data to high dimensional data. These could be employed as a pre-processing tool to reduce the dimensionality of the genomic data and reduce the computational burden of the method. We also anticipate significant gains in run times when the method is combined with variable screening. In this research, the performance was evaluated of the proposed method using metrics such as TPR and TNR to address the class-imbalance present. Balancing the class imbalance, prior to modeling, through techniques such as oversampling, under-sampling, and SMOTE (Chawla et al., 2002) can also be explored to improve the proposed three-stage method.

There is limited literature (Schrag et al., 2018; Akdemir et al., 2020; Lopez-Cruz et al., 2020; Arouisse et al., 2021; Sandhu et al., 2021) on combining data types to improve genomic selection. With the advances in modern plant breeding and access to an increasing number of data sources, it is essential to develop statistical approaches that will allow breeders to leverage all available data to improve selection strategies and accelerate breeding programs. Second, most of the focus over the past 2 decades has been on developing models for continuous traits that are normally distributed. Recently, there has been an increasing focus on non-Gaussian distributed traits. Our work simultaneously targets both these two gaps in the literature. Along with proposing approaches to combine data types, our methods rely on penalization and forward selection to reduce the model size. Breeders can use the methods to predict the categorical traits in their programs and more importantly, understand the impact of individual predictors on these categorical traits.

Finally, while we presented this method in an agronomic setting, the three-stage model proposed can be implemented in any problem involving combining data types of differing sizes. For example, we believe that it could be very useful in the area of precision medicine where the main trait is a risk of disease or reaction to a medication. The secondary traits could be other physiological measurements from the subjects, the medium-dimensional data could be the lifestyle and behavioral characteristics, and the high-dimensional data type could be the genomic marker information.

## Data Availability

The data analyzed in this study is subject to the following licenses/restrictions: The data set used in this study is available upon request. Requests to access these datasets should be directed to Reka Howard, rekahoward@unl.edu.
