# Supplementary material for: Integrating and optimizing genomic, weather, and secondary trait data for multiclass classification
Source: Front Genet. 2023 Mar 29;13:1032691. doi: 10.3389/fgene.2022.1032691 (PMC10090538; doi:10.3389/fgene.2022.1032691)
Supplement: Supplementary file 1 [file DataSheet1.pdf]

frontiers

Supplementary Material

1 FINLAY-WILKINSON REGRESSION

We find the optimal time windows for the environmental effect using a Finlay-Wilkinson (FW) regression-based approach. The mean of the primary trait for all genotypes in an environment represents the environmental covariate, which we will refer to as the environmental mean. A window of time for a weather variable is represented by the mean of all daily values of that variable in a given environment. For instance, suppose we have eight environments and 250 genotypes. Using the 250 genotypes, we compute the average trait value for each environment, giving us eight means. Then, suppose the window of time we are interested in is between day 10 and day 15 and the weather variable is wind speed. Then, we compute the average of all wind speeds between day 10 and day 15 for each environment, resulting in eight means again. Then, we fit a simple linear regression between these two sets of eight means to quantify the linear association between them using the R-squared value. We repeat this process over all possible time windows in the growing season and find the window with the highest R-squared value, i.e., the optimal time window. An example linear regression between the environmental means and the wind speed means is given in Fig. S1.

2 MULTI-CLASS CLASSIFICATION

One-vs-all (OVA) is the most straightforward approach to binarizing a K-class problem into separate binary classification sub-problems in such a way that each sub-problem utilizes a binary classifier to discriminate the kth class from the other $K-1$ classes. Thus, if we have K classes, we require K binary classifiers. To predict the class of a new observation, we predict the probability that it is class k using the kth classifier for each $k = 1, \ldots, K$ and assign that class associated with the largest probability.

One-vs-one (OVO) is another approach to creating binary classification sub-problems so that each sub-problem builds a discriminant to separate each pair of classes. If we have K classes, there are $\frac{K(K-1)}{2}$ pairs, and hence we require $\frac{K(K-1)}{2}$ binary classifiers. When classifying a new observation, we predict the class using each binary classifier and assign the class that is most frequent among the $\frac{K(K-1)}{2}$ classifiers.

3 CLASSIFICATION METRICS FOR BINARY TRAITS

Binary Classification

For binary classification, we evaluate the performance of models using metrics such as overall accuracy (Acc), true positivity rate (TPR), and true negativity rate (TNR). Overall accuracy is simply defined as the total number of correctly classified observations out of the total number of observations in the data set. While it is a simple and straightforward metric to understand, overall accuracy does not distinguish between the false positive and false negative types of error made. In other words, it does not differentiate between a 1 being classified as 0 from a 0 being classified as a 1. In situations where the false positive and false negative errors have differing consequences, accuracy may not measure the important characteristics. Further, overall accuracy does not signify the actual performance in the presence of class imbalance. Suppose we had a binary response with 995 observations in class 1 and 5 in class 0. A model can obtain an overall accuracy of 99.5% by simply classifying all observations as 1. Thus, metrics such as TPR and TNR

were defined to decompose the overall accuracy into accuracy by class to alleviate the problems described above. In binary classification, TPR, also known as sensitivity or recall, calculates the proportion of 1s that are actually classified as 1s. TNR, also known as specificity, refers to the proportion of 0s that are classified as 0s. Thus, we can evaluate the performance of the model for each class using TPR and TNR.

Using the confusion matrix in Fig S2, overall accuracy (Acc), TPR, and TNR can be defined as:

$$\text{Acc} = \frac{TP + TN}{N} \tag{S1}$$

$$\text{TPR} = \frac{TP}{TP + FN} \tag{S2}$$

$$\text{TNR} = \frac{TN}{TN + FP}, \tag{S3}$$

where TP was the number of true positives, TN was the number of true negatives, FP was the number of false positives, and FN was the number of false negatives. N was the total number of observations, which is also the sum of TP, TN, FP, and FN. Several other metrics have been developed for classification and a detailed review can be found in Tharwat (2020) and Grandini et al. (2020).

4 BINARY TRAIT IMPLEMENTATION

Days to Maturity (DM) is a continuous trait that quantifies the number of days from planting till the plant is mature enough to produce seeds. We discretized the DM trait into a binary variable with 0 indicating low and 1 indicating $high$ values to implement the binary classification algorithm. We used quantiles to separate the two classes as well as possible. For the continuous trait DM, we denoted all observations in the bottom 25th percentile as zeros and the top 25th percentile as ones.Changing the choice of percentiles would change the separation between the classes and impact the predictive ability of any classification method.

In order to evaluate the model thoroughly, we investigated its performance across several levels of class imbalance in the binary response. Class balance refers to a situation where the number of 1's and 0's in the response variable is approximately equal. In contrast, class imbalance refers to cases where one of the classes is more prevalent than the other in the response. Imbalanced response significantly impacts the predictive power of models, and so is an important consideration. Hence, we studied the stability of our method looking at increasing levels of imbalance. We looked at the model's performance under the following five ratios of 1's and 0's: 50-50, 65-35, 75-25, 85-15, 95-05. 50-50 refers to the case where the classes are balanced, while 95-05 refers to the most extremely imbalanced case where 95% of the observations are 1's and 5% are 0's. We randomly sampled 280 observations to create the datasets with different class ratios. For each class ratio, we created 20 replications and averaged the performance across the replications to avoid any bias due to the random sampling of the observations.

We split the 280 observations into training, optimization, and validation data sets in the ratio of 120/80/80, respectively. The training data set was used in the second stage, i.e., the main modeling step, to build the predictive model and obtain the coefficients associated with each predictor. The optimization data was used in the third stage of modeling to find the optimal threshold. Finally, the validation set was a new, previously unseen data set used to assess the model's performance. The coefficients from the training set and the

optimal threshold from the optimization set were combined to classify observations in the validation set. This approach ensures no information is shared between the different stages of the three-stage method proposed in this work. It guarantees the maximum generalizability of the model to predict the class of a new, previously-unseen observation with a high degree of certainty.

We evaluated the effect of penalization applied to the weather and genomic data types by comparing the performance and model size with a baseline model $G + E + P$ that had no penalty applied to any of the data types, denoted by M0. We also investigate case M1 where we included all the secondary traits in the final model (denoted by $PenG + PenE + P$), since the secondary traits were so few to start with. The main proposed model $PenG + PenE + PenP$, denoted by M2, has penalties applied to all three data types. Both the random forest and support vector machine models were fit on the entire predictor data set with no penalization of any sort. Table S1 summarizes all the model settings, including notation to represent the models in the results section.

5 RESULTS FOR BINARY TRAIT

We compared our proposed three-stage method to the RF and SVM methods. The proposed method had two primary objectives: improve predictive power and reduce model size. The model size refers to the number of variables with non-zero coefficients in the final model. The smaller the model size, the more interpretable the relationships are between the predictors and the binary response. In order to evaluate the predictive power, we employed TPR, TNR, and overall accuracy for a comprehensive study. First, we evaluated the models based on overall accuracy. All the proposed models showed an improvement in performance over the ML models in the balanced class setting. The baseline model (M0) had an accuracy of 0.73. The penalized versions (M1 and M2) showed an improvement over the baseline model with an accuracy of 0.75. In contrast, the RF only had a 0.68 accuracy, while the SVM model had the worst accuracy at 0.66. Thus, the proposed main model (M2) showed a substantial improvement of 12% in prediction accuracy over the ML methods. M1 and M2 had comparable performance with an increasing class imbalance in the response. The results can be seen in Fig. S3. One thing to note is the failure of every single prediction model in the extreme imbalance case of 95-05. In this situation, all models had a very ”high” accuracy score of close to 0.95. However, this is misleading since all models simply predict every observation as 1. Consequently, they end up with an accuracy of 0.95 since 95% of the observations are indeed in class 1. Across the board, the standard error associated with the average of the accuracy, TPR, and TNR were in the order of 10^{-3} to 10^{-4} and hence are not presented here.

The overall classification accuracy is an unreliable metric in the presence of class imbalance, as clearly evidenced in our results. Thus, we also examined the TPR and TNR values for the models in each class-balance setting. These results can be viewed in Figs. S4 and S5. For the TPR results in the balanced case, we again saw M1 and M2 significantly outperforming the ML methods. Interestingly, M1 and M2 had comparable or marginally better TPR values than RF and SVM across all class-balance settings.

Both M1 and M2 also showed improvement over ML methods in the TNR values for the balanced class with values of 0.73 as opposed to 0.64 for SVM and 0.65 for RF. However, the ML methods did have a more stable performance in the presence of class imbalance. In the extreme imbalance case, all methods have equally poor performance with all TNR close to 0 and RF with a TNR of exactly 0.

Equally crucial to the predictive performance of the models was model interpretability. Our methods showed a vast improvement over the ML methods in model sizes. SVM used all predictors for building the model and performing classification, while the RF model used several thousands of predictors. RF

had over 3500 variables in the balanced case, destroying any potential for interpretation of relationships or identifying key variables to classify the binary response. The baseline model (M0) had 250 variables across the different class balance settings. Penalization (M1 and M2) provided a massive improvement in the model sizes over the M0. The M1 model used 30.9 variables on average in the balanced class setting, while the M2 model used 26.3 variables on average. Thus, the penalized models required as few as 15% of variables as the baseline model. More importantly, the penalized models M1 and M2 had the best performance across all five models despite using the fewest variables. Fig. S6 visualizes the number of variables used by each model across the different class balance settings.

Looking deeper into the number of variables used by each model, we saw that none of the M0, M1, or M2 models selected any genomic variables directly. There may have been some genomic effect indirectly through the secondary trait residuals. All the variation in the response was explained by the secondary traits and the weather variables. In contrast, RF relied heavily on genomic information, as evidenced by the ratio of the selected genomic variables to the total number of variables. Across every class balance setting, over 90% of the variables selected by RF were genomic variables. Refer to Figs. S7 and S8 for further details. All the detailed results are summarized in Table S2.

6 SUPPLEMENTARY TABLES AND FIGURES

6.1 Figures

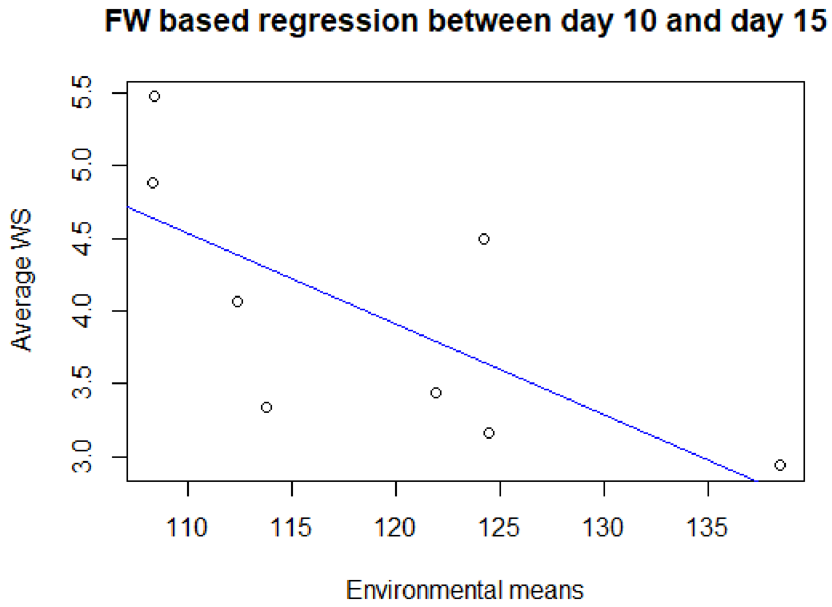

Figure S1. Linear regression between environmental means and mean wind speed for the eight environments. The points represent environments and the blue line represents the best-fit line from the linear regression.

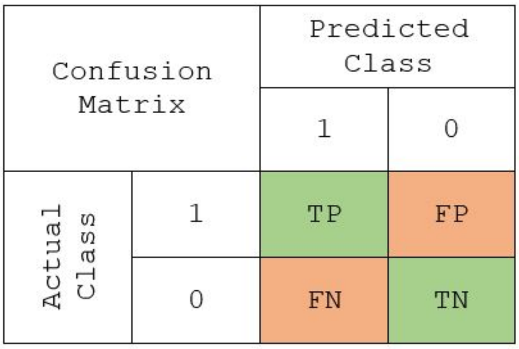

Figure S2. Confusion matrix for binary classification representing the true class vs predicted class: true positive (TP), false positive (FP), true negative (TN), and false negative (FN).

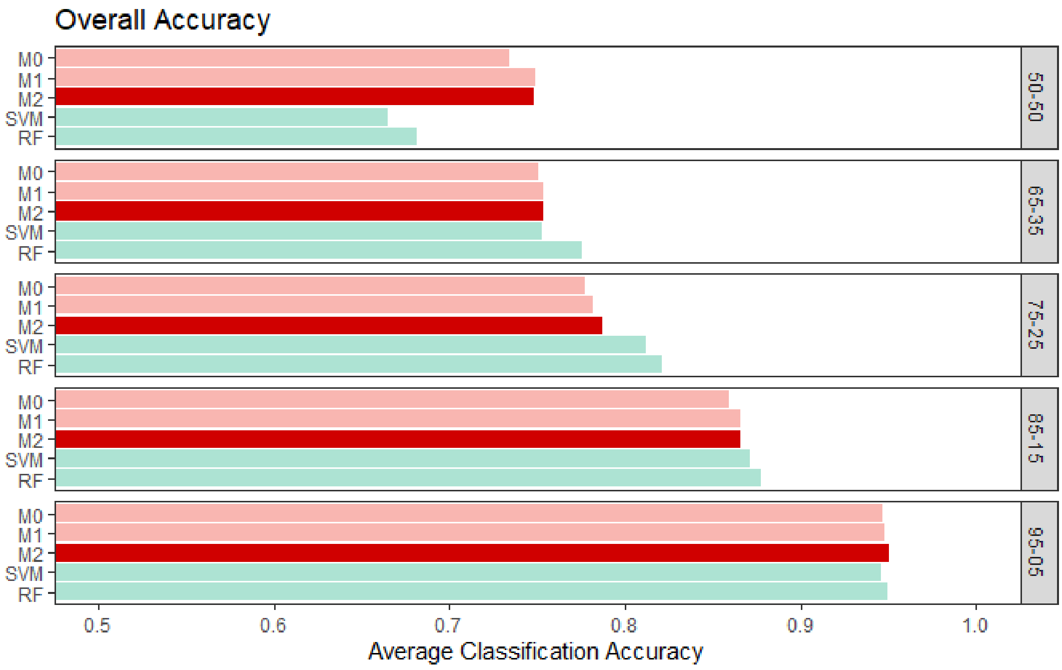

Figure S3. Bar plot comparing the 5 different classification models based on overall classification accuracy averaged over the 20 replications within each class balance setting for the binary trait. The five models and their notations are as follows: G + E + P (M0), PenG + PenE + P (M1), PenG + PenE + PenP (M2), support vector machine (SVM), and random forest (RF).

REFERENCES

Grandini, M., Bagli, E., and Visani, G. (2020). Metrics for Multi-Class Classification: an Overview. *arXiv:2008.05756 [cs, stat]* ArXiv: 2008.05756

Tharwat, A. (2020). Classification assessment methods. *Applied Computing and Informatics* 17, 168–192. doi:10.1016/j.aci.2018.08.003. Publisher: Emerald Publishing Limited

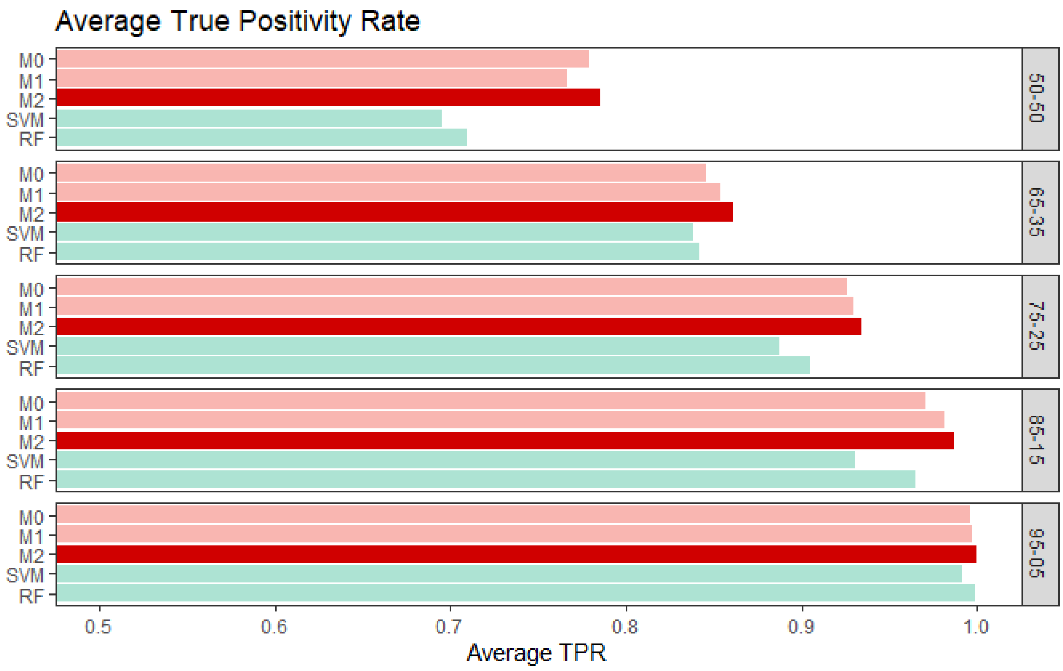

Figure S4. Bar plot comparing the 5 different classification models based on true positivity rate (TPR) averaged over the 20 replications within each class balance setting for the binary trait. The five models and their notations are as follows: G + E + P (M0), PenG + PenE + P (M1), PenG + PenE + PenP (M2), support vector machine (SVM), and random forest (RF).

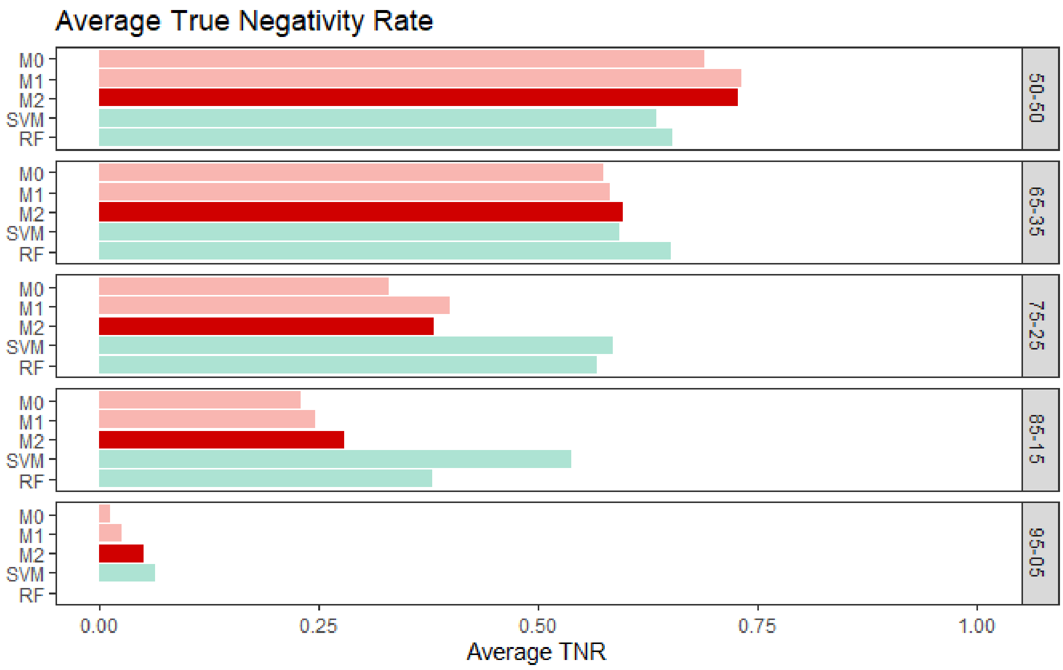

Figure S5. Bar plot comparing the 5 different classification models based on true negativity rate (TNR) averaged over the 20 replications within each class balance setting for the binary trait. The five models and their notations are as follows: G + E + P (M0), PenG + PenE + P (M1), PenG + PenE + PenP (M2), support vector machine (SVM), and random forest (RF).

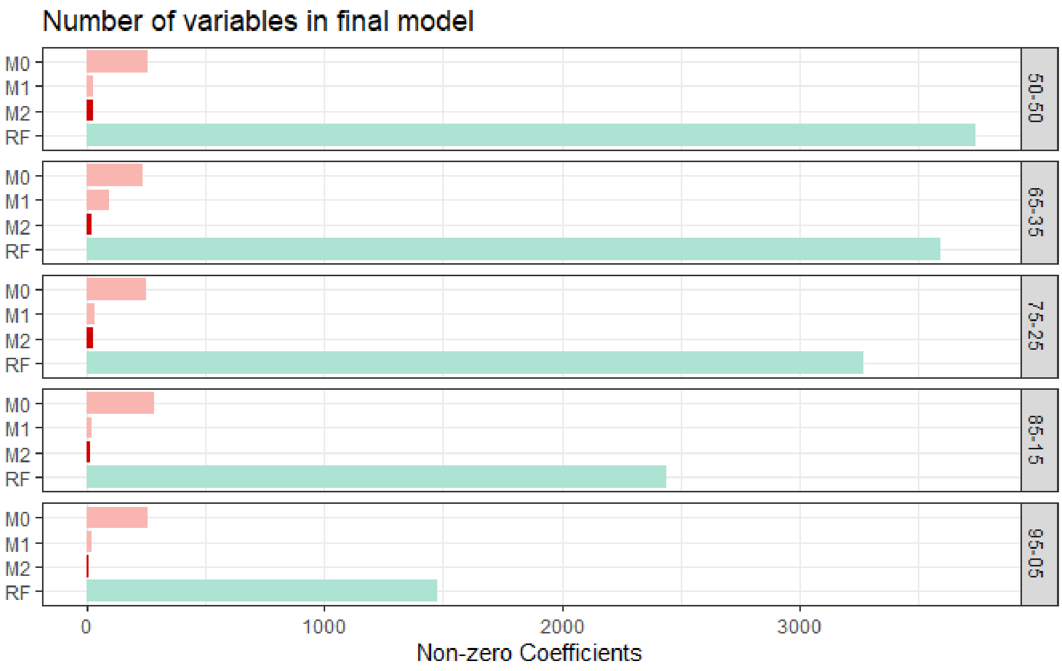

Figure S6. Bar plot comparing the proposed models to random forest based on model size averaged over the 20 replications within each class balance setting for the binary trait. The four models and their notations are as follows: G + E + P (M0), PenG + PenE + P (M1), PenG + PenE + PenP (M2), and random forest (RF).

Table S1. Summary of models assessed for the binary class trait

Model	Notation	λ_1	λ_2	λ_3
G + E + P	M0	0	0	0
PenG + PenE + P	M1	0	varying	varying
PenG + PenE + PenP	M2	varying	varying	varying
Support Vector Machines	SVM	-	-	-
Random Forest	RF	-	-	-

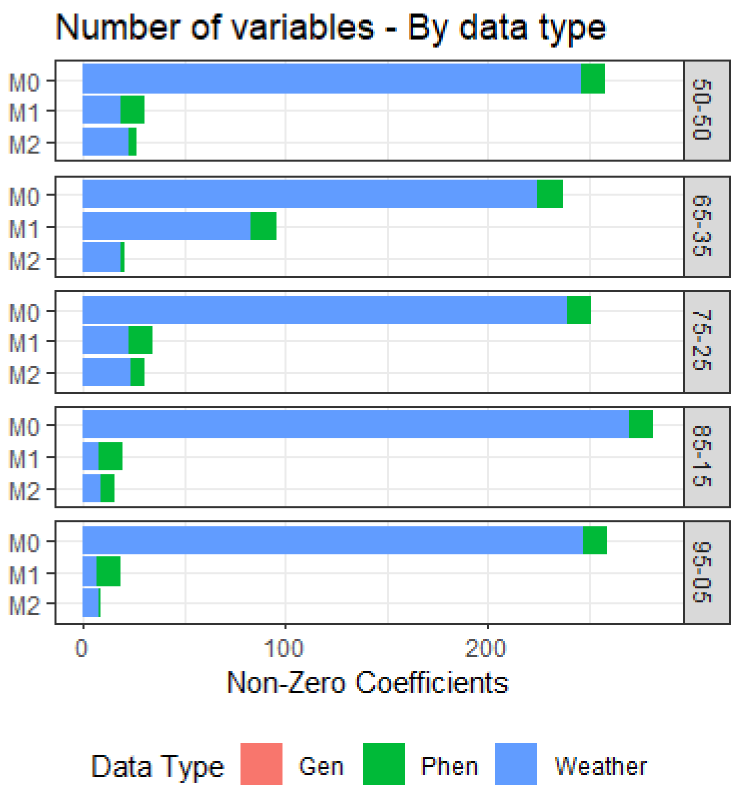

Figure S7. Bar plot comparing the proposed models based on model size partitioned by data type, averaged over the 20 replications within each class balance setting for the binary trait. The three models and their notations are as follows: G + E + P (M0), PenG + PenE + P (M1), and PenG + PenE + PenP (M2).

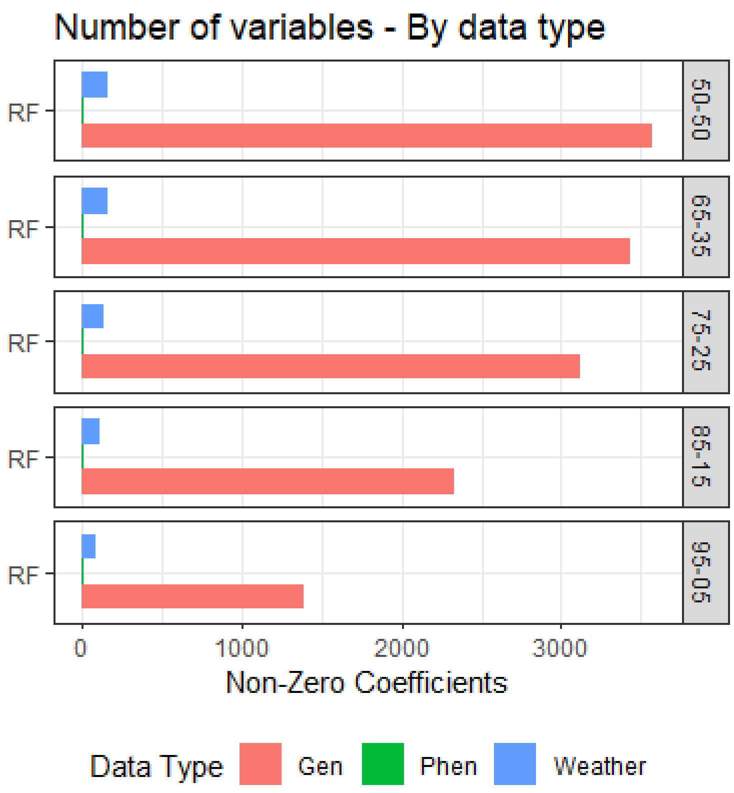

Figure S8. Bar plot for RF model sizes partitioned by data type, averaged over the 20 replications within each class balance setting for the binary trait.

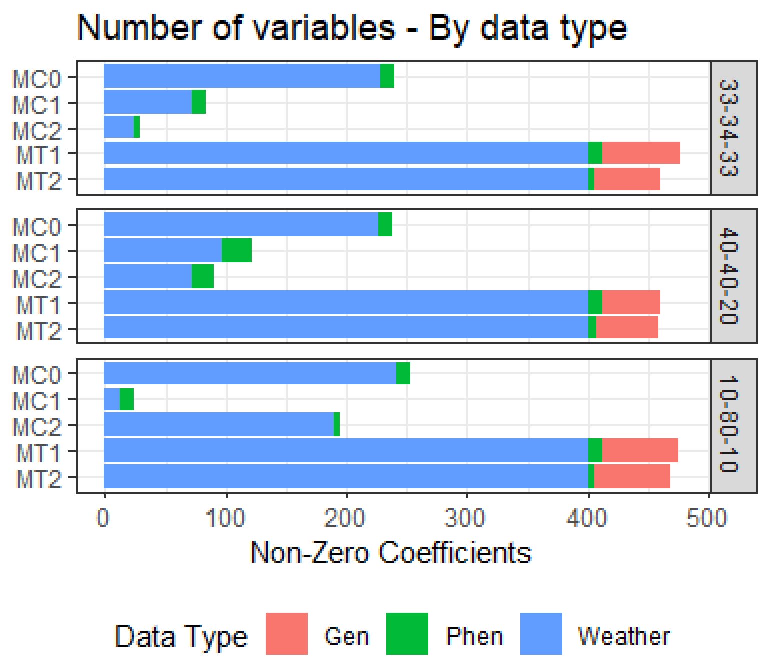

Figure S9. Bar plot comparing the proposed models based on model size partitioned by data type, averaged over the 20 replications within each class balance setting for the multiclass trait. The five models and their notations are as follows: G + E + P (MC0), PenG + PenE + P (MC1), PenG + PenE + PenP (MC2), PenG + FWE + P (MT1), and PenG + FWE + PenP (MT2).

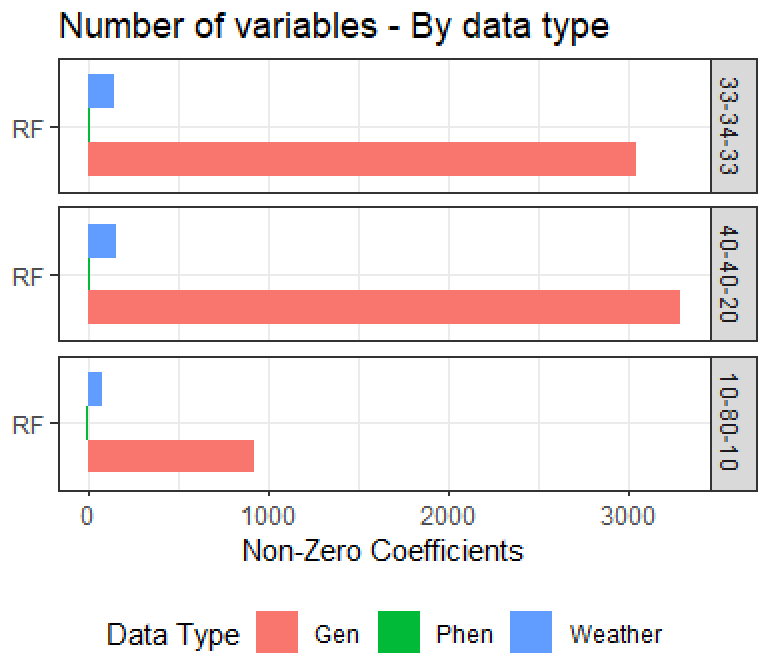

Figure S10. Bar plot for RF model sizes partitioned by data type, averaged over the 20 replications within each class balance setting for the multi-class trait.

Table S2. Summary of results for the five different models across the five different class-balance settings for the binary trait. The performance was measured using overall accuracy (Acc), true positivity rate (TPR), true negativity rate (TNR), and model size (MDS). The five models and their notations are as follows: G + E + P (M0), PenG + PenE + P (M1), PenG + PenE + PenP (M2), support vector machine (SVM), and random forest (RF).

Balance	Model	Acc	TPR	TNR	MDS
50 - 50	M0	0.73	0.78	0.69	258.9
	M1	0.75	0.77	0.73	30.9
	M2	0.75	0.79	0.73	26.3
	SVM	0.67	0.71	0.64	-
	RF	0.68	0.70	0.65	3740.8
65 - 35	M0	0.75	0.85	0.57	237.1
	M1	0.75	0.85	0.58	95.4
	M2	0.75	0.86	0.60	20.05
	SVM	0.75	0.84	0.59	-
	RF	0.78	0.84	0.65	3594.1
75 - 25	M0	0.78	0.93	0.33	251.3
	M1	0.78	0.93	0.40	34.4
	M2	0.79	0.93	0.38	30.5
	SVM	0.81	0.89	0.59	-
	RF	0.82	0.91	0.57	3267.5
85 - 15	M0	0.86	0.97	0.23	282.5
	M1	0.87	0.98	0.25	20.0
	M2	0.87	0.99	0.28	15.5
	SVM	0.87	0.93	0.38	-
	RF	0.88	0.97	0.54	2443.6
95 - 05	M0	0.95	1.00	0.01	259.0
	M1	0.95	1.00	0.03	18.4
	M2	0.95	1.00	0.05	8.3
	SVM	0.95	0.99	0.06	-
	RF	0.95	1.00	0.00	1477.2
